# Serine Proteases-Like Genes in the Asian Rice Gall Midge Show Differential Expression in Compatible and Incompatible Interactions with Rice

**DOI:** 10.3390/ijms12052842

**Published:** 2011-04-29

**Authors:** Deepak Kumar Sinha, Mulagondla Lakshmi, Ghanta Anuradha, Shaik J. Rahman, Ebrahimali A. Siddiq, Jagadish S. Bentur, Suresh Nair

**Affiliations:** 1 Plant Molecular Biology Group, International Centre for Genetic Engineering and Biotechnology, Aruna Asaf Ali Marg, New Delhi 110067, India; E-Mail: deepak22sinha@yahoo.co.in; 2 Acharya N. G. Ranga Agricultural University, Rajendranagar, Hyderabad 500030, India; E-Mails: lakshmi_1506@rediffmail.com (M.L.); saps_61@yahoo.com (G.A.); sjrahman1964@rediffmail.com (S.J.R.); easiddiq@rediffmail.com (E.A.S.); 3 Directorate of Rice Research, Rajendranagar, Hyderabad 500030, India; E-Mail: jbentur@yahoo.com

**Keywords:** biotype, chymotrypsin, insect-plant interaction, phytophagous insects, real time PCR, trypsin

## Abstract

The Asian rice gall midge, *Orseolia oryzae* (Wood-Mason), is a serious pest of rice. Investigations into the gall midge-rice interaction will unveil the underlying molecular mechanisms which, in turn, can be used as a tool to assist in developing suitable integrated pest management strategies. The insect gut is known to be involved in various physiological and biological processes including digestion, detoxification and interaction with the host. We have cloned and identified two genes, *OoprotI* and *OoprotII*, homologous to serine proteases with the conserved His^87^, Asp^136^ and Ser^241^ residues. *OoProtI* shared 52.26% identity with mosquito-type trypsin from Hessian fly whereas *OoProtII* showed 52.49% identity to complement component activated *C1s* from the Hessian fly. Quantitative real time PCR analysis revealed that both the genes were significantly upregulated in larvae feeding on resistant cultivar than in those feeding on susceptible cultivar. These results provide an opportunity to understand the gut physiology of the insect under compatible or incompatible interactions with the host. Phylogenetic analysis grouped these genes in the clade containing proteases of phytophagous insects away from hematophagous insects.

## 1. Introduction

The Asian rice gall midge, *Orseolia oryzae* (Wood-Mason) (Diptera: Cecidomyiidae), is the third most destructive insect pest of rice (*Oryza sativa* L.) with an average annual yield loss worth US $80 million in India alone [1]. It poses a serious concern in the rice growing regions of the world as it causes huge economic loss. The most effective method of managing the pest has been the development and deployment of resistant rice cultivars [2]. Owing to extensive cultivation of resistant cultivars the insect has been evolving into new biotypes [3] that are able to overcome the host resistance. Therefore, there is an urgent need to focus on and understand the interactions between the Asian rice gall midge and its host plant to aim at durable resistance against the pest.

Soon after hatching, the young larvae crawl onto the meristematic region near the crown of seedlings and commence feeding. The susceptible cultivars succumb to form tube-like galls—silver shoots—rendering the tiller sterile [1,4]. In the resistant cultivars, infestation leads to incompatible interaction resulting in insect mortality. The incompatible interaction manifests itself mainly as antibiosis in two distinct ways, *i.e.*, with or without the expression of hypersensitive response (HR) [5].

The gall midge-rice interaction operates on a gene-for-gene basis [6]. Anatomical, chemical and genetic studies of gall midge avirulence and plant resistance indicate that the plant defense is triggered by the interaction between the product of the avirulence (*avr*) gene of the gall midge and the product of the corresponding resistance (*R*) gene from the plant [7]. *Avr* typically segregates as a single dominant gene as well, while recessive mutation(s) within an avirulence locus (presumably the *avr* gene) allow insect survival [8]. A common feature of these *avr* genes is that they encode protein secreted into the host plants through various means [9] which contain factors determining virulence or avirulence. Insect salivary gland secretions contain many uncharacterized substance(s) including digestive enzymes that increase permeability of cell membranes and weaken host cell walls [10] aiding insect feeding and subsequent breakdown of the ingested plant material for absorption by the insect. Among the digestive enzymes present in the insect gut, serine proteinases (tyrpsins and chymotrypsins) are most abundant as also reported in the case of the Hessian fly (*Mayetiola destructor*: Diptera: Cecidomyiidae) [11].

Most host plants subjected to larval feeding produce proteinase inhibitors (PI), as inducible plant defenses that belong to different families of serine proteinase inhibitors [12]. PI produced by the plants lead to the starvation of the insects, due to its inability to digest plant proteins, and ultimately results in insect mortality. Although serine proteases have a highly conserved tertiary structural fold, they have developed a range of substrate specificities critical to many biological functions [13,14]. Mechanisms exist in insects that impart resistance to PI and include the upregulation of enzymes that degrade the PI [15], induction of enzymes that resist inactivation by PI [12,16], and overproduction of enzymes to maintain normal levels of gut proteolysis [17]. Owing to these observations, coupled with the fact that serine proteinases are the most abundant gut proteins in feeding larvae, it is hypothesized to play an important role in the interaction of the gall midge with its rice host.

Serine proteases play a vital role in digestion in the rapidly growing larvae and probably also have an important role to play in the gall midge-rice interaction. Currently, there is major headway being made with regard to the wheat-Hessian fly interaction [11]. However, in contrast, there are no reports of cloned genes encoding for digestive proteases from the rice gall midge. Besides, there is hardly any information on how these genes express in gall midges that are in a compatible or incompatible interaction with its rice host.

In the present study, we have investigated expression of the genes encoding digestive serine protease-like enzymes in the rice gall midge feeding on susceptible and resistant cultivars. As far as we know this is the first report of the cloning of genes that encode proteases in the rice gall midge and their differential expression in compatible and incompatible interactions. We believe that this study will be a step towards providing a better understanding of the interaction between rice gall midge and its host that would eventually ensure development of better strategies for protecting rice from this economically important pest.

## 2. Results and Discussion

Resistance against the Asian rice gall midge in rice is mainly expressed as larval antibiosis and is governed, generally, by a single dominant gene [18]. However, the molecular basis of this interaction between the gall midge and rice still needs to be deciphered.

When feeding on resistant cultivars, larvae are killed within 24–96 h. This could be brought about by the inability of the larvae to digest and/or detoxify the ingested plant material. Therefore, we thought it pertinent to investigate the significance of gut proteases having a role in this interaction as suggested in the case of the Hessian fly, a midge of wheat and another member of the family Cecidomyiidae [11].

**Figure 1.**
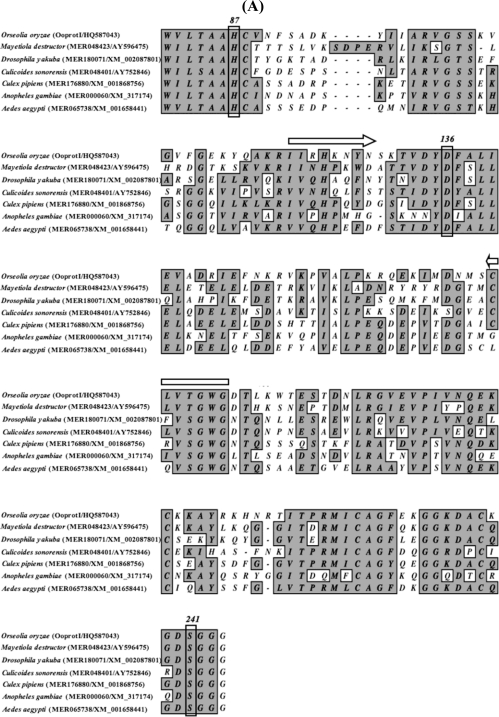

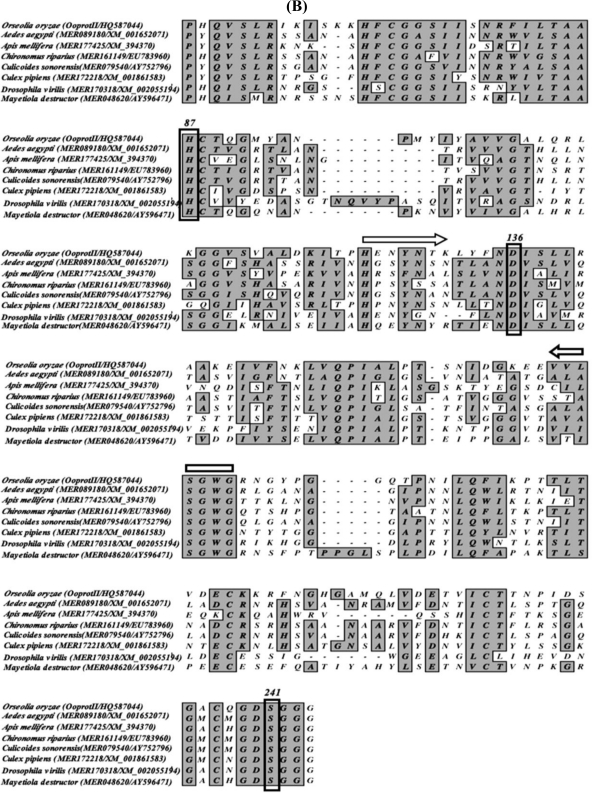
Multiple sequence alignment of OoprotI (**A**) and OoprotII (**B**) of *Orseolia oryzae* showing homology to different insects. Conserved amino acid residues are highlighted and active sites are boxed. Horizontal arrows show the regions from which the RT-PCR primers were made.

**Figure 2.**
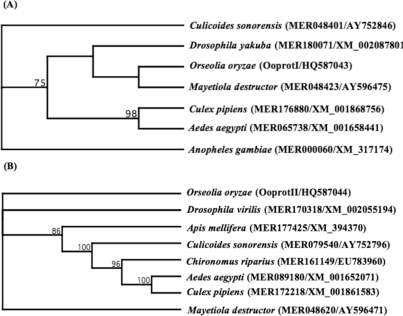
Phylogenetic tree derived from homology between insect trypsins and OoprotI (**A**) and insect chymotrypsins and OoprotII; (**B**) The phylogenetic tree was constructed using 1000 replications. Figures at nodes represent bootstrap values above 50%. Numbers in brackets are MEROPS IDs followed by GenBank Accession numbers. The branch lengths are arbitrary. The tree was constructed using the Neighbor-joining method and pair-wise distances were calculated using Poisson-corrected distance method included in the MacVector suite of programs.

Here we report, for the first time, the cloning and transcriptional expression patterns of two serine protease-like genes (designated as *OoprotI* (Acc. No. HQ587043) and *OoprotII* (Acc. No. HQ587044)) from the Asian rice gall midge. BLAST searches of the amino acid sequences inferred from RNA sequence data revealed that *OoProtI* has 52.26% identity with mosquito-type trypsin from *M. destructor* (Hessian fly). *OoProtII* showed 52.49% identity to complement component activated *C1*s from the Hessian fly [calcium-dependent serine proteinase, C1 esterase (MER048620)]. The classification of these genes was primarily based on the identity shared at the amino acid level with those from other dipterans. Further, the conserved amino acid residues were correctly positioned with reference to the other previously identified proteases and, therefore, likely to confer serine proteases-like specificity to the enzymes (Figures 1A and 1B). Within the predicted amino acid sequences of *OoProtI* and *OoProtII*, the active site triad (Histidine-87, Aspartic acid-136, Serine-241) was conserved. Phylogenetic analysis (Figure 2A) revealed that *OoProtI*, coding for a trypsin-like protein, was the closest in terms of homology to that of the Hessian fly. *OoProtII* (Figure 2B), in terms of phylogeny, falls in a completely different group of its own, while all the proteases of the hematophagous insects are grouped into a single clade. Importantly, in this phylogenetic grouping, other than *Apis*, all the non-hematophagous insects fall into separate groups *i.e.*, *Drosophila*, *Mayetiola* and *Orseolia*. This type of a grouping is expected as proteases of phytophagous insects and those from the hamatophagous insects differ probably in the substrate amino acid constituents on which respective enzymes have to act.

**Figure 3.**
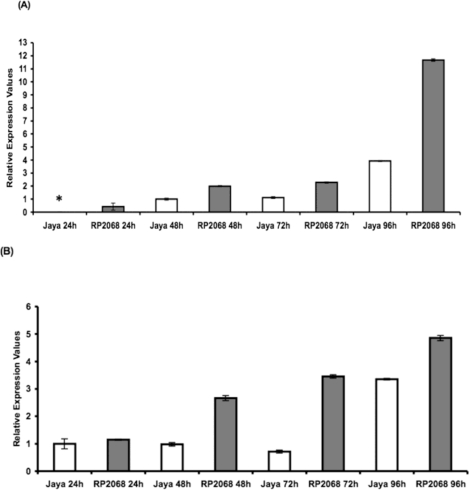
Transcript levels of *OoprotI* (**A**) and *OoprotII* (**B**) of the rice gall midge feeding on compatible host Jaya (white bars) and incompatible host RP2068 (black bars) 24, 48, 72 and 96 h after hatching. Two biological replicates and two technical replicates were taken for the study. Asterisk (*) indicates no detectable amplification. Error bars represent mean ±SD.

Based on the quantitative Real Time (RT) PCR we observed that both *OoProtI* (Figure 3A) and *OoProtII* (Figure 3B) expressed in the larvae examined at 48, 72 and 96 h post hatching, independent of the fact that they were feeding on the resistant (RP2068) or the susceptible (Jaya) plants with highest expression observed at 96 h. However, the expression level of *OoProtI* was three-fold higher at 96 h when feeding on a resistant cultivar compared with larvae feeding on the susceptible cultivar. In the case of the expression pattern of *OoProtII*, we observed a similar pattern. Here too the expression of *OoProtII* was the highest at 96 h and the expression of this transcript was approximately one and a half times higher at 96 h in the larvae feeding on the resistant cultivar as compared to larvae on the susceptible cultivar. It should be noted that in the case of *OoProtI,* we did not observe any amplification in 24 h post-hatch larvae even after repeating the PCR amplifications on several batches of larvae from this stage. Owing to this reason, amplification at 48 h time point was used as calibrator for *OoProtI* but for the case of *OoProtII* 24 h post-hatch time point was used as the calibrator. It may be noted that trypsins hydrolyze peptide bonds involving amino acids with positively charged side chains, such as arginine and lysine, whereas chymotrypsins cleave peptide bonds on the carboxyl terminus of aromatic amino acids (tryptophan, tyrosine and phenylalanine) [19]. It has also been reported that functional diversity of encoded enzymes and differential ability to hydrolyze ingested proteins may account for the regulatory dexterity evident from differential gene expression of multiple, sequence divergent midgut proteases [20]. The observation that expression levels of both *OoProtI* and *OoProtII* were the highest at 96 h and when feeding on the resistant cultivar, could be due to the response of the larvae to the possible presence of PI and/or other toxins. The PI could be inducing their up-regulation and/or the *de-novo* synthesis promoting changes in key amino acids that help these two proteases resist the inhibitors [21]. However, this needs to be confirmed. Although both *OoProtI* and *II* were overexpressed in larvae feeding on the resistant cultivar, the larvae eventually succumb.

It has been suggested that resistance of insects to PI is based, at least in part, on their ability to enhance the proportion of “inhibitor-resistant” enzymes in the midgut. The susceptibility of an insect to a PI is directly related to the proportion of proteolytic enzyme activity in the midgut that can be suppressed by that inhibitor [12]. We believe that this study will be a step towards providing a better understanding of the interaction between rice gall midge and its host that will eventually ensure development of better strategies for protecting rice from this economically important pest. Further, data reported here will provide us with an alternative strategy to enhance resistance in host plants in addition to the presently available method of deploying “R” genes for resistance leading to/resulting in a dual-pronged approach for achieving lasting/durable resistance to gall midge in rice.

## 3. Experimental Section

### 3.1. Insect Material

A colony of gall midge biotype 4 (GMB4) insects was maintained in the greenhouse at the Directorate of Rice Research, Hyderabad, India, on a susceptible indica rice cultivar as described [3]. Rice seedlings (15-day-old) of both Jaya (susceptible) and RP2068-18-3-5 (resistant) cultivars were infested with GMB4. GMB4 is virulent on rice cultivar Jaya and avirulent on RP2068-18-3-5 (henceforth referred to as RP2068) possessing resistance gene *gm3*.

### 3.2. Collection of Larvae and RNA Isolation

Rice seedlings (15-day-old) of both Jaya and RP2068 variety were infested with GMB4 and regularly monitored as described earlier [3]. Larvae were dissected out from individual rice seedlings under the microscope, at different time intervals of 24, 48, 72 and 96 h after egg hatching. The larvae were collected in RNAlater (Ambion, USA) and stored at −80 °C until utilized. Approximately 600 larvae each (from both cultivars) were dissected out. RNA was isolated using RNeasy Plus Micro Kit (Qiagen GmbH, Germany) following the manufacturer’s protocol. Two temporally separated biological replicates were included for the studies.

### 3.3. cDNA Library Preparation

Total RNA was isolated from 100 larvae each from the four time points *i.e.*, 24, 48, 72 and 96 h. Equal amount of RNA (quantitated using NanoVue spectrophotometer (GE Healthcare, U.K.)) was pooled and SMART pico cDNA synthesis kit (Clontech, USA) was used for library construction following the manufacturer’s protocol. cDNA inserts were cloned directly into PCR4-TOPO vector (Invitrogen, USA). Plasmid DNA was commercially sequenced by Macrogen Inc, South Korea.

### 3.4. Sequence and Phylogenetic Analysis

Vector contamination was removed and the raw sequences were analyzed using MacVector (v11.1.2; MacVector Inc., USA) using the in-built phred-phrap algorithm. Sequence similarity analysis was performed using the NCBI-BLAST program (http://blast.ncbi.nlm.nih.gov). MEROPS (http://merops.sanger.ac.uk), the peptidase database was used to identify the specific MEROPS family/subfamily and the percentage identity of the sequences obtained in this study with those in the database.

A phylogenetic tree was constructed using the amino acid sequence of serine proteases of other insects so as to reveal the relationship of the genes isolated in this study with its homologues. The tree was calculated by Neighbor-joining method available in the MacVector suite of programs. Bootstrap values for the branches were obtained with 1000 replications. Poisson-corrected distances were accounted for the phylogeny and the gaps were distributed proportionally. Phylogenetic tree was also constructed using the nucleotide sequences (data not shown) of nearest organism taking into account the distances after Tamura-Nei correction.

### 3.5. RT-PCR and Statistical Analyses

The Primer Express Software 3.0 (Applied Biosystems, USA) was used to design the RT-PCR primers for *OoprotI*, *OoprotII* and control genes. Equal quantity of total RNA (as estimated by NanoVue), from different time intervals, were reverse transcribed using Superscript III reverse transcriptase enzyme (Invitrogen, USA) to single stranded cDNA using oligo dT primers following the manufacturer’s protocol. Equal amounts of cDNA (22 ng) were taken for all the RT-PCR analyses. Twenty μL of PCR mix contained 1X Power SYBR Green PCR mix (Applied Biosystems, USA) and 0.5 μM of the primers. The cycling conditions were: 95 °C for 10 min followed by 40 cycles of 95 °C for 15 s and 60 °C for 1 min. The primer pairs used were RTOoprot IIF: TCCCCAGCCAGACAAAACA and RTOoprot IIR: CACGAAAACTACAACACCAAGC; RTOoprot IF: CCCAACCAGTCACCAAACA and RTOoprot IR: TTCGGCACAAAAACTACAATTCA; RTactinF: TGAGACACCATCACCGGAATC and RTactinR: ATCCAAAGGCCAATCGTGAA. Quantification of mRNA levels were displayed as Relative Expression Values (REV) based on relative standard curve method (StepOne Real-Time PCR System, Applied Biosystems, USA). Results were analyzed using 2^−^^ΔΔ^^*C*^*T* method built into the StepOnePlus Real-Time PCR analysis software (Applied Biosystems, USA) provided with the instrument. Two biological replicates and two technical replicates were taken for the study. Actin was chosen as the internal control for all the RT-PCR assays reported here as it was found to be the most stable housekeeping gene under our experimental conditions. We evaluated, using geNorm software [22], three other genes, GAPDH, Elongation Factor 1-α, and Ribosomal Protein 17 before choosing actin as internal control.

## 4. Conclusions

In summary, we report here for the first time the cloning and sequencing of genes coding for digestive serine protease-like enzymes from the rice gall midge. Using RT-PCR, the expression pattern of these two genes was revealed. Importantly, expression of these genes was higher in larvae feeding on the resistant cultivar than those feeding on the susceptible cultivar. Thus, the two genes may play a significant role in the interaction of the rice gall midge with the host plant.
